# Periorbital Purpura as a Key Diagnostic Clue in Immunoglobulin Light Chain Amyloidosis

**DOI:** 10.31662/jmaj.2024-0357

**Published:** 2025-03-07

**Authors:** Kazuki Miyaue, Hiroki Isono

**Affiliations:** 1 Department of General Medicine, HITO Medical Center, Ehime, Japan

**Keywords:** amyloidosis, periorbital purpura, raccoon eyes, heart failure, kidney failure

A 55-year-old man presented with exertional dyspnea and bilateral leg swelling lasting several months. On examination, bilateral periorbital purpura (Figure 1) and pretibial pitting edema were noted. Laboratory testing showed elevated creatinine and brain natriuretic peptide. Serum immunofixation electrophoresis revealed lambda-type M protein. Echocardiography indicated biventricular hypertrophy with diastolic dysfunction. Biopsies of the skin and rectal mucosa showed Congo red-positive amyloid deposits (Figure 2) and apple-green birefringence under polarized light microscopy (Figure 3). Based on clinical and pathological findings, a diagnosis of immunoglobulin light chain (AL) amyloidosis was made. AL amyloidosis is a systemic condition characterized by amyloid deposits in various organs, leading to multiorgan dysfunction ^(1)^. Early symptoms are typically nonspecific, which often delays diagnosis and exacerbates organ damage ^(2)^. Periorbital purpura, commonly referred to as raccoon eyes, is rare but highly specific to AL amyloidosis ^(1)^. Its presence was a key diagnostic clue, leading us to an early suspicion of AL amyloidosis. In this case, alternative eyelid conditions, such as atopic eczema, ocular rosacea, and contact dermatitis, were less likely given the lack of pertinent medical history, the absence of lid margin telangiectasias or conjunctival injection, and the absence of pruritus or other skin manifestations.

**Figure 1.**
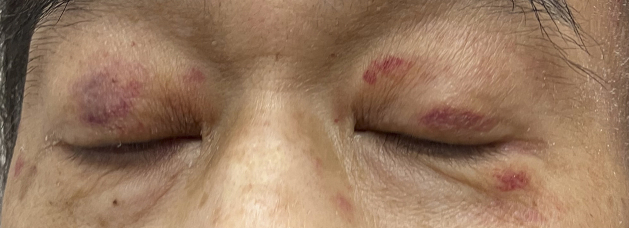
Picture of the patient’s face showing bilateral periorbital purpura.

**Figure 2.**
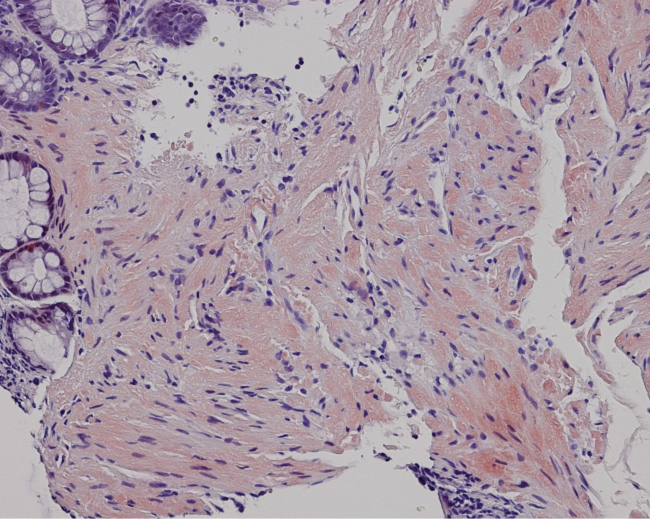
Biopsy specimen of rectal mucosa showing amorphous extracellular material with positive Congo red staining.

**Figure 3.**
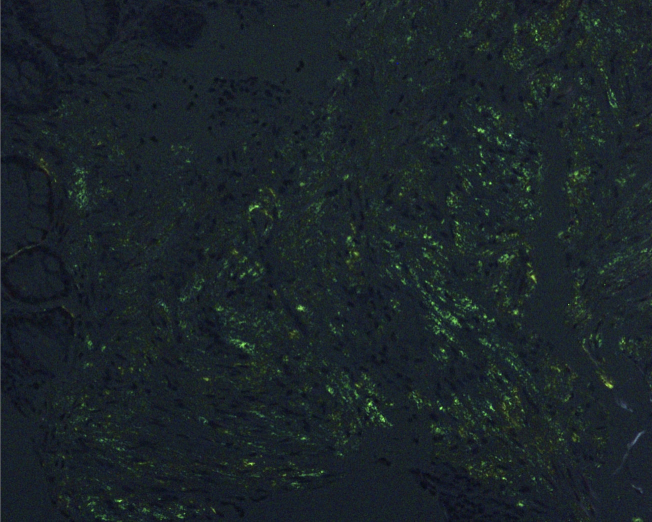
Biopsy specimen of rectal mucosa showing apple-green birefringence under polarized light microscopy.

## Article Information

### Conflicts of Interest

None

### Author Contributions

Kazuki Miyaue and Hiroki Isono were involved in the conception or design of the work, drafting the work or reviewing it critically for important intellectual content, final approval of the version to be published, and agreement to be accountable for all aspects of the work in ensuring that questions related to the accuracy or integrity of any part of the work are appropriately investigated and resolved.

### Approval by Institutional Review Board (IRB)

Approval from the ethical board was not required.

### Informed Consent

Consent to publish the details of the present case was obtained from the patient.
